# Environmental Enrichment for Broiler Breeders: An Undeveloped Field

**DOI:** 10.3389/fvets.2017.00086

**Published:** 2017-06-09

**Authors:** Anja B. Riber, Ingrid C. de Jong, Heleen A. van de Weerd, Sanna Steenfeldt

**Affiliations:** ^1^Department of Animal Science, Aarhus University, Tjele, Denmark; ^2^Wageningen Livestock Research, Wageningen, Netherlands; ^3^Cerebrus Associates Ltd., The White House, Godalming, United Kingdom

**Keywords:** behavior, broiler breeder, genotype, environmental enrichment, welfare

## Abstract

Welfare problems, such as hunger, frustration, aggression, and abnormal sexual behavior, are commonly found in broiler breeder production. To prevent or reduce these welfare problems, it has been suggested to provide stimulating enriched environments. We review the effect of the different types of environmental enrichment for broiler breeders, which have been described in the scientific literature, on behavior and welfare. Environmental enrichment is defined as an improvement of the environment of captive animals, which increases the behavioral opportunities of the animal and leads to improvements in biological function. This definition has been broadened to include practical and economic aspects as any enrichment strategy that adversely affects the health of animals (e.g., environmental hygiene), or that has too many economic or practical constraints will never be implemented on commercial farms and thus never benefit animals. Environmental enrichment for broiler breeders often has the purpose of satisfying the behavioral motivations for feeding and foraging, resting, and/or encouraging normal sexual behavior. Potentially successful enrichments for broiler breeders are elevated resting places, cover panels, and substrate (for broiler breeders housed in cage systems). However, most of the ideas for environmental enrichment for broiler breeders need to be further developed and studied with respect to the use, the effect on behavior and welfare, and the interaction with genotype and production system. In addition, information on practical use and the economics of the production system is often lacking although it is important for application in practice.

## Introduction

Broiler breeders are commonly housed in barren environments and subjected to feed restriction, especially during rearing, giving cause to a wide range of welfare problems [e.g., Ref. (1, 2)]. Severe feed restriction is implemented in the conventional production of broiler breeders to reduce the occurrence of health and reproduction problems that would occur if the birds were fed *ad libitum*. However, paradoxically, feed restriction itself is the source of many welfare problems observed in these birds [e.g., Ref. (3)]. Unfulfilled behavioral needs and hunger are common (2), resulting in birds showing signs of stress (4–7) and performing behavior indicative of frustration, boredom, and hunger [see review by D’Eath et al. (8)].

The growth potential of genotypes used in organic broiler production (9), or so-called “middle segment” broiler systems (10), may differ. Therefore, for some breeder genotypes there is a continued need for a certain degree of feed restriction, whereas for other genotypes no feed restriction (or for only one of the sexes) is required (11). Although organic production systems are often richer in stimuli, a number of welfare problems can still arise under these conditions (12).

Aggression is a major welfare problem, both between roosters and between hens in the competition for feed (13) and especially during sexual behavior throughout the production period (14, 15). Sexual behavior in broiler breeders lacks many of the elements of normal sexual behavior, which both jungle fowl and laying hens show (15–17). Millman et al. (15) compared layer and broiler breeder males and found that the latter displayed higher levels of sexual aggression. Since sexual aggression was not affected by feeding regime, they concluded that the differences between the two strains were associated with genetic factors. Broiler breeder males often force matings, which results in stress and fear in hens (15–18). In production systems, roosters typically stay in the littered area while hens tend to stay at the raised slats (18). This decreases the number of mating possibilities resulting in increased competition among the roosters, which in turn can result in forced mating and the hens being exposed to stress and damage to plumage and skin. Beak trimming is often performed to reduce plumage and skin damage, caused by rough mating and feather pecking. Also, despurring and toe clipping, only performed in males, are used as preventive measures to reduce scratches and wounds inflicted by males to hens during mating. In some European countries, mutilations (i.e., beak trimming, despurring, and toe clipping) in broiler breeders are—or will be—banned (e.g., Sweden and The Netherlands) (19), calling for development of new effective methods to prevent damaging behavior in broiler breeders.

A frequently quoted definition by Newberry (20) states that environmental enrichment is a modification of the environment of captive animals, thereby increasing behavioral possibilities and leading to improvements of the animal’s biological function. Enriched environments accommodate a larger range of behavioral choices (21), and it has been suggested that this can lead to a reduction in welfare problems in all types of production systems (22). Environmental enrichment is provided with the purpose of (1) increasing the occurrence of the animal’s species-specific behavior, (2) preventing the development of abnormal behavior or reducing its extent and complexity, (3) increasing the positive exploitation of the environment (e.g., the use of an outdoor area), and (4) increasing the animal’s ability to handle behavioral and physiological challenges (20). In addition, environmental enrichment must be biologically relevant to be effective. Van de Weerd and Day (21) broadened Newberry’s definition, to include practical and economic aspects of enrichment. This takes into consideration that any enrichment strategy that adversely affects the health of animals, or that has too many economic or practical constraints will never be implemented on commercial farms and thus never benefit animals (Ibid.). For example, broiler breeder production imposes severe demands on environmental hygiene, which can be prohibitive for implementing certain types of enrichment, such as bales of straw.

The aim of this paper is to give an overview of the different kinds of environmental enrichment applied during the rearing and laying periods for broiler breeders, as described in the scientific literature. Furthermore, the purpose is to assess the effects of the enrichment on behavior and welfare according to Newberry’s (20) definition, as well as Van de Weerd and Day’s (21) thesis.

## Methods

Relevant scientific literature was retrieved from the database “Web of Science” using the key words: “broiler breeder environmental enrichment.” In addition, the paper includes studies identified on reference lists of papers reviewed and conference abstracts. Only peer-reviewed papers were included (in English or German). The time-frame criteria for inclusion were that sources were published in or after the year 2000. Because of intensive genetic selection in broilers and breeders (19, 23), the interactions between enrichment, behavior, and welfare found in older references may not be of relevance to modern day birds.

### Assessing Effectiveness

Each enrichment has been described in terms of the resource it provides (inputs) and in terms of its effects on the birds (outcomes). A range of indicators specifically for broiler breeders can be applied when assessing welfare outcomes (1, 24). In this paper, we have mainly focused on assessing the effects of environmental enrichment on stereotypies (pecking objects, excessive drinking behavior, and pacing), the type of mating (forced, interrupted, or successful), time spent on feeding, foraging, and resting on elevated places, level of aggression as well as damage to the skin, keel bone, and plumage. There are other relevant indicators that could be used to assess the effect of enrichment on broiler breeder welfare, e.g., the occurrence of courtship behavior, feather pecking, fear, growth, and body weight. However, we did not find studies that measured these indicators in relation to environmental enrichment.

## Environmental Enrichment for Broiler Breeders

Four refereed papers and three conference abstracts met the search criteria stated in the methods and were therefore included in the study. The limited literature mainly regards parent animals of conventional broilers while literature on parent animals of slower growing broiler genotypes (e.g., organic or middle segment broilers) is very sparse. Only point-source enrichment in standard environments has been studied. Point-source enrichments are objects (such as bales or elevated resting places) added to a house or pen in a conventional production system, with the purpose of enhancing the environment (21). The objects are generally limited in size, and their use is often restricted to a single, or a few, location(s). Table 1 provides an overview of the design of the point-source enrichment objects used in the different studies: elevated resting places, cover panels, bales of wood shavings, suspended strings, materials for foraging and dustbathing, and feed scattering.

**Table 1:** Perch, platform, panel, substrate bales, strings, substrate, and feed scattering designs for broiler breeders used in different studies.

Reference	Material	Period	Design			Genotype		Stocking density
			Height above ground	Dimensions (length × width × depth)	Style and placement	Slow growing	Fast growing	
**Perches and platforms**								
(26)	Elevated slats Grids above feeders Plastic beams	Production	N/A	N/A	–	JA	Ross^a^	N/A

(27)	Wooden beams	Rearing and production	25, 50, 75, and 100 cm 55, 68, 115, and 138 cm	N/A	Aerial perches Aviary tiers	Sasso	Ross 308	N/A

**Panels**								
(18)	Frame: wooden bars, mesh: black plastic and chicken wire	Production	–	70 cm × 10 cm × 70 cm	Center of house	–	Roaster line^a^	6.7 birds/m^2^

**Substrate bales**								
(13)	White wood shavings	Rearing	–	79 cm × 38 cm × 28 cm	Plastic covered, corners	–	Ross 308	10.7 birds/m^2^

(28)	Wood shavings	Rearing	–	N/A	Plastic covered	–	Ross 308	N/A

**Strings**								
(13)	White polypropylene strings	Rearing	Top just above bird’s head	16 cm long	2 locations (center and wall)	–	Ross 308	10.7 birds/m^2^

**Substrate**								
(29)	Wood shavings	Rearing	–	N/A (depth)	Entire pen	–	Hubbard S	N/A

**Feed scattering**								
(30)	Standard commercial feed	Rearing	–	–	Entire pen	–	Hybro G	2.7 birds/m^2^

*N/A, information not available*.

*^a^Genotype not specified further*.

### Elevated Resting Places

Elevated resting places can be used for resting during day- and night-time. The environment in broiler breeder production houses is often more complex than in rearing houses; as a minimum there are elevated nest boxes that the hens must learn to access. Water nipples are often placed above elevated slats forcing both roosters and hens to move in the three-dimensional space. Provision of elevated resting places, particularly during rearing, promotes the development of the birds’ three-dimensional use of the production houses. Early access to perches has been shown to have a positive effect on the development of spatial cognitive skills in laying hens (25).

Gebhardt-Henrich and Oester (26) examined the use of elevated resting places (raised slats and grids above the feed trough) during the night in both the rearing and production periods in broiler breeders of the fast-growing genotype Ross and the slow-growing genotype JA (genotypes were not further specified). In general, JA made better use of the elevated resting places (91% of the birds at 20 weeks of age), but Ross also used these frequently (80% at 20 weeks of age). JA continued the high use with age, but for Ross, use decreased to around 50% at 53 weeks of age. Gebhardt-Henrich and Oester (26) also provided perches to Ross broiler breeders with continued access to elevated resting places through grids above the feed troughs and raised slats. The pattern of use with age corresponded with the previous study. The perches were only used to a low extent (<1%), probably because these were lower than the other elevated resting places, of which the slats were used most frequently followed by the grids above the feed troughs. Results on other welfare indicators were not presented for this study.

Gebhardt-Henrich et al. (27) also examined the use of perches during the rearing and production periods in broiler breeders of the fast-growing genotype Ross 308 and a slow-growing Sasso genotype. Two types of perch configurations above the slats were studied: eight aerial perches (14 cm of perching space per bird), or four aviary tiers with perches, all at different heights. The birds preferred the perches over the elevated structures present in all pens (grill over feeders, slats, and tube above drinker), and the four-tier configuration had more birds perching. Production was not impaired. Keel bone fractures were seen significantly more often in birds with access to perches (levels around 26–32%), and Sasso birds (39%) had higher levels than Ross birds (15%). Plumage condition was better in birds with access to the four-tier configuration than in the eight perch configuration and better in Sasso than in Ross birds. This study suggests that for roosting at night, broiler breeders prefer perches over slats and tubes above drinkers, and perches on aviary tiers over aerial perches.

### Cover Panels

Leone and Estevez (18) examined the effect of providing panels in the litter area during the production period in broiler breeders (roaster line). Their hypothesis was that the panels could function as a kind of shelter to avoid aggressive interactions and repeated mating of the same hen. The panels would result in better distribution of the roosters in the house and attract the hens to the littered area and alleviate stress in the hens, thus potentially improving reproduction. In total, 20 panels were provided on the littered area (390 m^2^) centrally placed in the house. Hens with panels in the house laid more eggs. The number of fertilized eggs from these hens peaked later and at a higher percentage. Finally, hatchability decreased less with age in hens with panels meaning that the effect of the panels increased with age. The home range of the roosters was larger for roosters in houses with panels, and these roosters stayed more in the area with slats than did the roosters from the control treatment. No observations of mating behavior, damage to the hens, or the hens’ use of the littered area were recorded.

### Bales of Wood Shavings and Strings

In rearing houses with feed-restricted conventional breeder pullets (Ross 308), Hocking and Jones (13) allocated bales of wood shavings wrapped in plastic and bunches of plastic strings to stimulate explorative pecking and foraging behavior. The plastic was removed when the hens had destroyed the bales. The bales were replaced when the breeders were 6 weeks old and after this, at least every second week. There were three treatments: no enrichment, enrichment from day 0, and enrichment from 8 weeks of age. Purposes were to examine whether the birds would use the two types of enrichment and to examine the effect on the level of aggressive pecks and the plumage condition. The use of strings was estimated from the condition of the string bunches scored at 8 and 18 weeks of age on a 5-point scale, whereas the use of bales was recorded as the number of birds associated with (not further defined) the bales at 5, 10, and 16 weeks of age. Aggression was defined as a vigorous peck aimed at the head, comb, neck, or back that resulted in the immediate withdrawal of the recipient, usually with accompanying vocalization, a similar aggressive threat or chasing.

The use of the strings was limited; however, these were used more by birds getting access to the enrichment when 8 weeks old, compared to those getting access from day 0 onward. The use of the bales was also rather limited and decreased with age. There was no difference in use of the bales at 10 and 16 weeks of age (either provided at day 0 or at 8 weeks of age). The proportion of birds pecking at walls and litter decreased with age, whereas pecking at drinkers increased, suggesting that the bales did not influence stereotypic pecking. No effect of allocation of strings and bales was found on the level of aggression or condition of the plumage (13). However, King (28) did find an effect on aggression by providing bales of wood shavings in deep-litter pens (1 bale per 100 birds), as Ross 308 birds significantly reduced aggressive head pecks (not defined further) by 40% in the late rearing phase (18 weeks of age). Birds used the bales to perch, peck, and forage.

### Provision of Materials for Foraging and Dustbathing

Hocking et al. (29) examined whether rearing of feed-restricted breeder pullets (Hubbard S) on wood shavings or slatted floor, respectively, had an effect on behavior. At 8 weeks of age, the two treatments were divided into four so that the groups either continued with the same treatment or received the other treatment.

At 4 weeks of age, birds on wood shavings performed more foraging behavior, less feather pecking, fewer stereotypic pecks at the wall and feeder, and fewer aggressive pecks [defined as in Hocking and Jones (13)]. The number of stereotypic pecks at the walls at 10 weeks of age continued to be highest in birds housed on slatted floors during the entire rearing period. In addition, at 10 weeks of age more plumage damage was observed in birds reared on slatted floors.

Globally, the majority of broiler breeders are housed during the rearing period with a combination of a litter- and a raised slatted-floor area (19). However, in some countries broiler breeders are housed in (enriched) cages, mainly during the production period (Ibid.). The results from this study emphasize the importance of providing foraging and dustbathing material.

### Scattering of Feed in the Bedding

De Jong et al. (30) studied the effect of scattering all feed in the bedding for conventional breeder pullets (Hybro G). This feeding method is used by some producers of broiler breeders to stimulate foraging behavior. Stereotypic pecks on objects (i.e., the cage, but not drinking nipple or litter) were reduced, but no other indicators of hunger (concentration of plasma corticosterone, compensatory food intake, and plasma glucose:non-esterified fatty acids ratio) were influenced by feed being scattered either in the bedding or provided in the feed trough.

### Summarizing Table

Table 2 summarizes the use and effects of the different environmental enrichments that have been studied on the prevalence of different welfare and production indicators measured.

**Table 2:** A summary of broiler breeders’ use of the different kinds of environmental enrichment reviewed in the present review and its effect on the prevalence of different welfare and production indicators measured.

Type	Period	Genotype	Use by the birds	Parameter affected					
				Stereotypic behavior	Sexual behavior	Plumage condition	Aggressive pecking	Keel bone damage	Other
Elevated resting places (perches and platforms)	Rearing	Fast	Limited						
		Slow	Moderate						
	Production	Fast	Moderate			Improved		Increased^a^	
		Slow	Well used			Improved		Increased^a^	
Cover panels	Production	Fast							Improved^b^
Bales of wood shavings	Rearing	Fast	Limited	No effect		No effect	Reduced		
Suspended strings	Rearing	Fast	Limited			No effect	No effect		
Substrate	Rearing	Fast	Well used	Reduced		Improved	Reduced		
Scattering of feed in bedding	Rearing	Fast		Reduced					No effect^c^

*Fast, fast growing, slow, slow growing*.

*^a^Only studied in birds with access to both perches and platforms*.

*^b^Egg production, fertility, hatchibility, and distribution of roosters in the house*.

*^c^Concentration of plasma corticosterone, compensatory food intake, and plasma glucose: non-esterified fatty acids ratio*.

## Discussion

According to Newberry’s (20) and Van de Weerd and Day’s (21) definitions, environmental enrichment should promote species-specific behavior and prevent the development of abnormal behaviors. Both vertical panels in the litter area, as well as elevated resting places, seem promising enrichments in that they promoted species-specific behavior as well as production. In addition, elevated resting places reduced plumage damage. However, the effect of panels on sexual behavior, plumage and skin damage in hens and the distribution of hens in the house need further study. Knowledge on the effects of providing elevated resting places for broiler breeders is also limited, but it is considered to be a type of enrichment that may have a positive effect on behavior and welfare. The design of elevated resting places should be optimized, to offer maximum use, but without negative side effects such as keel bone damage. Provision of elevated resting places seems to have no effect on production parameters, such as fertility, but further studies are needed.

Broiler breeders are subjected to feed restriction, especially during rearing, leading to stress, frustration, and hunger, and resulting in stereotypic object pecking, overdrinking, hyperactivity, and aggression [e.g., Ref. (1, 2)]. Providing substrate significantly reduced stereotypic pecking and aggression (29) and can thus be considered successful enrichment for cage-housed breeders. However, aggressive behavior and stereotypic pecking were not reduced when enrichment such as bales of wood shavings, strings, or feed scattering were applied in birds housed on litter floors (13, 30), and the use of bales and strings appeared to be limited (13). Feed restriction can be considered a major stressor (8), and it can be questioned whether abnormal behaviors due to feed restriction can be alleviated by providing environmental enrichment that stimulates explorative behavior. Providing diluted diets (low energy and high-fiber diets) to feed-restricted broiler breeders seems more promising in terms of improving welfare (2) than only providing enrichment that stimulates foraging behavior in littered houses. Similarly, pregnant sows are feed restricted and for this reason EU legislation (EU Council Directive 2008/120/EC) requires the provision of a sufficient quantity of bulky or high-fiber food to satisfy their hunger and need to forage. Whether enrichment such as bales of wood shavings, strings, or feed scattering are more successful in other types of broiler breeder production systems, using slower growing genotypes or diluted diets, remains to be studied.

A new commercial housing system has been introduced in The Netherlands, in which broiler breeder males and females are separated for 5 h a day using separate feeding systems and a movable partition (31). Experiments showed more voluntary and successful matings, as well as improved sexual behavior and better plumage condition of the females. Separating the sexes for a few hours per day seems to have a promising reducing effect on aggressive behavior of the males (Ibid.). It would be interesting to study different enrichments in these systems to further improve welfare and health of the broiler breeders, as well as economics and practical application.

While there are a number of studies available on the use of environmental enrichment for laying hens [e.g., Ref. (32–34)] and broiler chickens [see review by Riber et al. (submitted)^1^], there are very few studies on environmental enrichment for broiler breeders. These few studies indicate that there are possibilities for successful enrichment that may promote species-specific behavior and thus welfare of broiler breeders. Environmental enrichment that stimulates foraging behavior and/or improves mating behavior is especially relevant with the discussion in some European countries toward phasing out mutilations of broiler breeders. More research into effective environmental enrichment for broiler breeders is needed to improve the welfare of these birds, both in conventional and alternative production systems.

## Author Contributions

AR conceived the idea of the mini-review. The manuscript was prepared and edited by all the authors.

## Conflict of Interest Statement

The authors declare that the work was conducted in the absence of any commercial or financial relationships that could be construed as a potential conflict of interest.

## Footnotes

^1^Riber AB, Van de Weerd HA, de Jong IC, Steenfeldt SS. Review of environmental enrichment for broiler chickens. (submitted).
